# Supplementary material for: Altered microRNA Transcriptome in Cultured Human Liver Cells upon Infection with Ebola Virus
Source: Int J Mol Sci. 2021 Apr 6;22(7):3792. doi: 10.3390/ijms22073792 (PMC8038836; doi:10.3390/ijms22073792)
Supplement: Supplementary file 1 [file ijms-22-03792-s001.zip › Supplementary_File/D_ KEGG_Analysis_Results/16-30nt_pathway_Makona-96h-Huh7_vs_Control-96h-Huh7_down.mature_mirna_targets/hsa_EnrichmentScore.pdf]

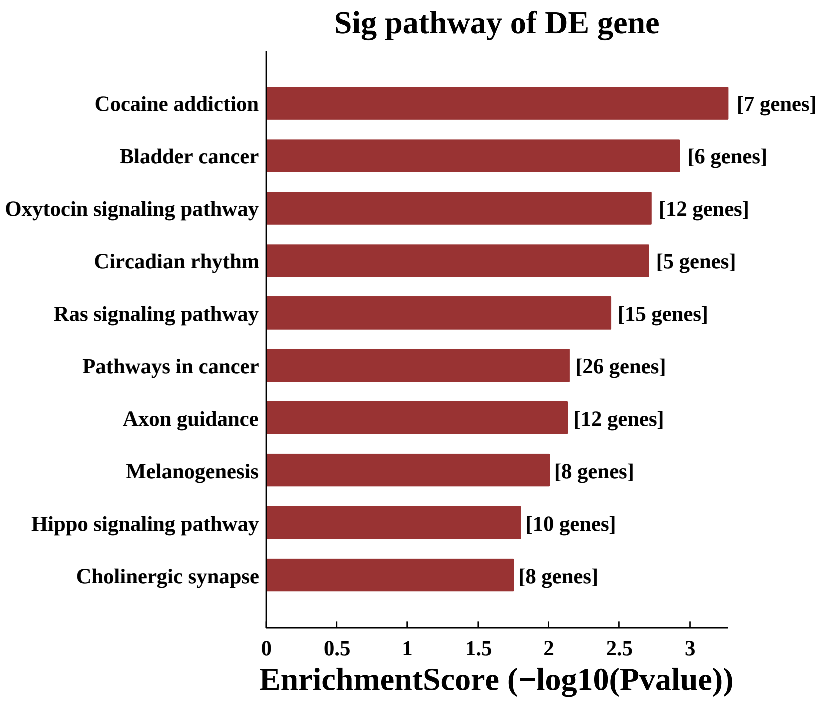
Sig pathway of DE gene
Cocaine addiction
Bladder cancer
Oxytocin signaling pathway
Circadian rhythm
Ras signaling pathway
Pathways in cancer
Axon guidance
Melanogenesis
Hippo signaling pathway
Cholinergic synapse
[7 genes]
[6 genes]
[12 genes]
[5 genes]
[15 genes]
[26 genes]
[12 genes]
[8 genes]
[10 genes]
[8 genes]
0
0.5
1
1.5
2
2.5
3
EnrichmentScore (−log10(Pvalue))
